# Supplementary material for: Association between Conflicts of Interest Disclosure and Quality of Clinical Practice Guidelines in Japan: A Meta-Epidemiological Study
Source: J Pers Med. 2023 Dec 17;13(12):1722. doi: 10.3390/jpm13121722 (PMC10744963; doi:10.3390/jpm13121722)
Supplement: Supplementary file 1 [file jpm-13-01722-s001.zip › jpm-2756405-supplementary.pdf]

Supplement Table S1. Items for reporting methodological research, adapted from the PRISMA Checklist

Section/topic	#	Checklist item	Reported on page #
TITLE			
Title	1	Identify the report as a meta-epidemiologic study.	1
ABSTRACT			
Structured summary	2	Provide a structured summary that includes the background of the topic, goal of the study, data sources, method of data selection, appraisal and synthesis methods, results, limitations, conclusions and implications of key findings.	2,3
INTRODUCTION			
Rationale	3	Describe the rationale for the meta-epidemiological study in the context of what is already known.	5
Objectives	4	Provide an explicit statement of the goal of the meta-epidemiological study and the hypothesis being empirically tested.	6
METHODS			
Protocol	5	Indicate if a protocol exists, if and where it can be accessed (eg, Web address). Registration of a protocol is not mandatory	7
Eligibility criteria	6	Specify study characteristics used as criteria for eligibility with a rationale.	7
Information sources	7	Describe all information sources (eg, databases with dates of coverage, contact with experts to identify additional studies, Internet searches) and search date.	8-10
Search	8	Present full electronic search strategy for at least one database, including any limits used, such that it could be repeated. Search is commonly not driven by a clinical question.	NA
Study selection	9	Describe the process for selecting studies for inclusion (ie, how many reviewers selected studies, reviewing in duplicate or by single individuals).	7

Data collection process	10	Describe method of data extraction from reports (eg, piloted forms, independently, in duplicate) and any processes used for manipulating data or obtaining and confirming data from investigators.	8-10
Data items	11	List and define all variables for which data were sought and any assumptions and imputations made.	11,12
Risk of bias in individual studies	12	If risk of bias assessment of individual studies was relevant to the analysis, describe the items used and how this information is to be used during data synthesis.	NA
Summary measures	13	State the principal summary measures (eg, ratio of risk ratios, difference in means) and explain its meaning and direction to readers.	12
Synthesis of results	14	Describe the statistical or descriptive methods of synthesis including measures of consistency if relevant. If applicable, describe the development of statistical or simulation modelling based on theoretical background. Describe and justify assumptions and computational approximations. Describe methods of additional analyses (eg, sensitivity or subgroup analyses, meta-regression), if done, indicating which were prespecified.	12
RESULTS			
Study selection	15	Give numbers of studies assessed for eligibility and included in the study, with reasons for exclusions at each stage, ideally with a flow diagram. Present a measure of inter-reviewer agreement (eg, kappa statistic).	12
Study characteristics	16	For each study, present characteristics for which data were extracted and provide the citations. Clinical characteristics may not always be relevant.	12
Risk of bias within studies	17	If risk of bias assessment of individual studies was used in the meta-epidemiological analysis, report risk of bias indicators of each study to allow replication of findings.	NA
Results of individual studies	18	Present data elements used in the meta-epidemiological analysis from each study (results of clinical outcomes may not be relevant).	NA
Synthesis of results	19	Present results of statistical analysis done, including measures of precision and measures of consistency. Present validity of assumptions and fit of statistical or simulation modelling, if applicable.	14

Additional analysis	20	Give results of additional analyses, if done (eg, sensitivity or subgroup analyses, meta-regression).	14
DISCUSSION			
Summary of evidence	21	Summarise the main findings and compare them with existing knowledge about the topic. The quality of evidence may not be relevant; however, investigators should describe their certainty in the results to readers.	14
Limitations	22	Discuss limitations at research methodology level (eg, likelihood of reporting or publication bias).	17
Conclusions	23	Provide general interpretation of the results and implications for future research. Provide any plausible impact on clinical practice.	17
FUNDING			
Funding	24	Describe sources of funding for the methodology research and role of funders.	19

From: Murad MH, Wang Z. Guidelines for reporting meta-epidemiological methodology research. Evid Based Med. 2017 Aug;22(4):139–42.

Supplement Table S2. Included 23 Japanese CPGs

Name of main committing Japanese medical societies	n
Japanese Orthopaedic Association	6
Japanese Dermatological Association	4
Japan Gastroenterological Endoscopy Society	2
Japan Society of Medical Oncology	2
The Japanese Continence Society	2
Japan Diabetes Society	1
Japan Geriatrics Society	1
Japan Society of Neurology	1
Japan College of Rheumatology	1
Japanese Society of Cancer Nursing	1
Japanese Society for Radiation Oncology	1
Japanese Urological Association	1

Supplement Table S3. Detailed data of financial conflicts of interest from drug companies for the CPG developers in 23 Japanese CPGs published in 2019

	2016			2017			2018			2019			2020		
	Writing	Speaking	Consulting	Writing	Speaking	Consulting	Writing	Speaking	Consulting	Writing	Speaking	Consulting	Writing	Speaking	Consulting
CPG developers with payments	18	160	50	17	172	41	18	158	43	9	158	49	7	124	35
CPG developers with payments > $ 5,000	14	149	48	14	159	38	12	129	39	7	152	47	6	113	30
CPG developers with payments > $ 10,000	5	115	26	2	119	14	2	113	15	1	100	15	1	73	8
CPG developer	0	8	0	0	8	0	0	8	0	0	2	0	0	2	0

s with payments > $100,000

CPG, clinical practice guideline.
